# Ethical, legal and social aspects of the approach in Sudan

**DOI:** 10.1186/1475-2875-8-S2-S3

**Published:** 2009-11-16

**Authors:** Badria B El Sayed, Colin A Malcolm, Ahmed Babiker, Elfatih M Malik, Mohammed AH El Tayeb, Nageeb S Saeed, Abdel Hameed D Nugud, Bart GJ Knols

**Affiliations:** 1 Tropical Medicine Research Institute, National Centre for Research. P.O. Box 1304, Khartoum, Sudan; 2 School of Biological Sciences, Queen Mary, University of London, UK; 3 National Malaria, Schistosomiasis & Leishmaniasis Administration, Federal Ministry of Health. P.O. Box 1204, Khartoum, Sudan; 4 Sudan Atomic Energy Commission, Ministry of Science and Technology, P.O. Box 3001, Khartoum, Sudan; 5 National Health Laboratory, Ministry of Health, P.O. Box 287, Khartoum, Sudan 1; 6 Div. Infectious Diseases, Tropical Medicine & AIDS, Academic Medical Center, F4-217, Meibergdreef 9, 1105 AZ Amsterdam, The Netherlands and K&S Consulting, Kalkestraat 20, 6669 CP Dodewaard, The Netherlands

## Abstract

The global malaria situation, especially in Africa, and the problems frequently encountered in chemical control of vectors such as insecticide resistance, emphasize the urgency of research, development and implementation of new vector control technologies that are applicable at regional and local levels. The successful application of the sterile insect technique (SIT) for the control of the New World screwworm *Cochliomyia hominivorax *and several species of fruit flies has given impetus to the use of this method for suppression or elimination of malaria vectors in some areas of Africa including Northern State of Sudan. The research and development phase of the Northern State feasibility study has been started. Sudanese stakeholders are working side-by-side with the International Atomic Energy Agency in the activities of this important phase. Several ethical, legal and social issues associated with this approach arose during this phase of the project. They need to be seriously considered and handled with care. In this paper, these issues are described, and the current and proposed activities to overcome potential hurdles to ensure success of the project are listed.

## Background

The global burden of disease and death related to malaria remains a major concern in many parts of the world. Current estimates by the World Health Organization indicate that malaria infects 300-500 million cases worldwide, with at least one million deaths every year [1]. Despite the great efforts to control this disease, malaria remains a pubic health problem in more than 90 countries inhabited by more than 40% of the global population. The percentage of the world's population at malaria risk reached 48% in 2002 [2]. More than 90% of all malaria cases occur in sub-Saharan Africa, where in some areas over 70% of the population is continuously infected with the most deadly form of the parasite, *Plasmodium falciparum*. Malaria is endemic in the whole of Sudan, ranging from hypo-endemic in the north to hyper- and holo-endemic in the south. Transmission is seasonal (following the rainfall) in most of the country, and perennial in irrigated areas adjacent to the River Nile [3]. It is highly prevalent among children and pregnant women. It accounted for 37.2% of all hospital maternal deaths in Sudan [4]. Malaria is hypo-endemic in areas adjacent to the Nile in the Northern State, and represented 26.8% of 17 hospitals' outpatients in 2007, with a peak of 40 8% recorded in August [5]. *Anopheles arabiensis *is the only vector in Northern State where its efficacy as a malaria vector is due to its preference for human blood meals and its rapid adaptation to changes in the environment induced by human habitation and agriculture [6,7].

The emergence of problems associated with malaria treatment and control, such as drug and insecticide resistance, have led to a search for alternative control measures. Therefore the integration of the sterile insect technique (SIT) in an area-wide integrated pest management (AW-IPM) programme for malaria vectors is being considered. The Sudan Government has requested support from the International Atomic Energy Agency (IAEA) and the Islamic Development Bank (IDB) for the application of the SIT in Northern State to help eliminate the population of *An. arabiensis *from this region. This region fulfils certain criteria that make it suitable for an AW-IPM programme incorporating the release of sterile males [8]. In spite of several SIT trials carried out on mosquitoes ([9-13] and [14]), this is the first time that a malaria vector is being targeted on an area-wide basis using the SIT in sub-Saharan Africa. Targeting a public health problem like malaria gives this SIT feasibility study special relevance. The ethical, legal and social aspects of the approach and its potential economic impact are highlighted here.

## The study area

The ecological nature of the Northern State is highly unusual and is one reason why it was chosen for this feasibility study [8]. The residential area is confined to a narrow strip along the River Nile, extending from Egypt in the north up to the fourth cataract in the south. This strip is surrounded by the Great Desert on the eastern and western sides. The total population of about 600,000 is located in small villages close to the banks of the River Nile. The residents are primarily of Nubian and Arabian origin belonging to three main tribes. The State has a desert climate with only occasional rain. The maximum temperature in summer may reach 47°C during May, and in winter (December-February) the minimum temperature may drop as low as 7°C. Residents are mainly dependent economically on agriculture, which is based on irrigation and influenced by flooding of the Nile and seasonal changes in temperature. Cultivation is dominated by date palms but wheat and beans are cultivated in winter and maize and sorghum in summer.

## Stakeholders

**Figure 1:**
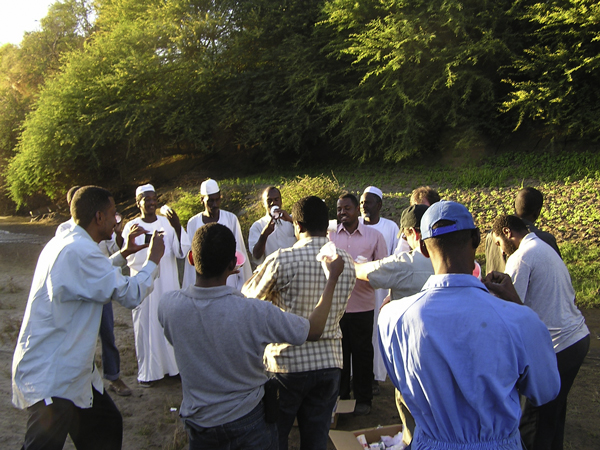
**Stakeholders performing the first release of sterile males of *Anopheles arabiensis *in Northern Sudan**. In addition to local leaders, representatives from the Tropical Medicine Research Institute, National Malaria Control Programme, National Health Laboratory, Northern State Ministry of Health, International Atomic Energy Agency, CDC Atlanta, Queen Mary College, the London School of Hygiene and Tropical Medicine are participating in the event.

The development of an AW-IPM programme for mosquito including SIT requires great national and international cooperative efforts drawing on stakeholders, partners and communities, each with a specific role and contribution. The programme will integrate larval control and residual house spraying with the release of sterile male mosquitoes. The programme requires that the partners are able and willing to address the legal, ethical and social concerns that arise. Commonly involved in these programmes are multiple sponsors, international agencies and the national government. The different stakeholders with their differing perspectives, interests and capacities need to be fully involved with all aspects of programme planning, implementation and evaluation in an ethically acceptable manner to ensure benefits for the community. Therefore, it is essential to build healthy relationships among all parties (Figure 1).

Strong links and good communication among the involved institutions are essential for project success. Inclusion of the Sudan Atomic Energy Commission (SAEC) as a strong national link between the IAEA and the project leaders within the stakeholders will facilitate the smooth progress of the project. Moreover, the SAEC regulates acquisition and use of irradiators containing radioactive materials and provides technical training as needed.

Dealing with an important public health problem like malaria necessitates the involvement of both the Federal and State ministries of health. Understandably, many activities related to the development of the site for the SIT feasibility study will benefit the current malaria control activities, such as detailed information on location of breeding sites and improved malaria diagnosis. Better information gathering, distribution, and coordination of activities will have a short-term benefit in reducing malaria transmission. In turn, the SIT would benefit by being able to release sterile insects when the wild mosquito population is low and when there is an improved malaria situation.

Since a geographic information system (GIS) is an essential component of the current project [15], national scientists and research institutions with good facilities and experience in remote sensing, GIS and topography must be involved. In Sudan particularly, other international agencies including the World Health Organization, the Global Fund, Roll Back Malaria, and the joint Egypt-Sudan Gambiae Control Project in Northern State are also involved. National ministries and agencies are also numerous: The Ministries of Science and Technology, Health, Finance, Irrigation and specific agencies including the Malaria Administration, the Dam Implementation Unit, the Meteorology Department, and Remote Sensing Authority. Joining the current effort with these institutions will help the feasibility study to achieve its objectives. Finally, a competent scientific research institute, the Tropical Medicine Research Institute, with its good infrastructure is hosting the project and is responsible for supervising and executing all the national research and development activities.

Political support and commitment of the government are key issues in the implementation of this feasibility study; this has been achieved from the beginning and will continue. Aside from the necessary assent to conducting the project, private and government entities have provided funds for field trips, vehicles, field stations and personnel. This ensures smooth implementation and execution of the different phases of the project. It also encourages and motivates the public to accept the idea of the project and to participate in its implementation. Also the project should have access to relevant information present in different government departments, and through this support can make use of existing facilities. In addition, political stability and healthy relationships with the public, press and political entities are essential to sustain control efforts [14].

## Ethical aspects

When the relevant health and research institutions began to develop the feasibility study, they gave immediate attention to informing people how a successful project would help in eliminating the malaria vector. In addition, it was made clear that other benefits resulting from the research and development would be made available readily and affordably to the stakeholders and the whole country. These include improved malaria diagnostic techniques and laboratory capabilities including equipment and better trained technicians and scientists.

Malaria is a public health problem in Northern State, affecting its economy and development. Although the total population of the state is not large, much money is spent currently on malaria treatment and control. If it is assumed, conservatively, that the above-mentioned 17 hospitals' outpatients represent the state population, elimination of the malaria vector will protect about 186,000 individuals from being infected every year. With limited resources assigned to the programme for continuing surveillance following elimination of the vector, the area could be maintained free of malaria vectors in a sustainable manner. Therefore, the implementation of such a project in an endemic area like Northern State will have a very positive impact on all the stakeholders.

The socio-economic impact of the feasibility study is expected to be great. The current cost of treatment of an uncomplicated malaria case in Sudan averages US$ 12 [16]. More than US$ 2,232,000 is spent annually in Northern State for malaria treatment. In addition, the vector control programme consumes more than 17 tons of insecticides, and the high running cost is about US$ 260,000 per year (E. M. Malik, personal communication). Therefore, a successful feasibility study will have a major economic impact, enhance the development of the area, and minimize harmful effects on human health and the environment by reducing the need for chemical control of the vector.

In spite of these obvious benefits, some aspects of the approach may still be a cause for concern. Some may be concerned about the effects of eradicating the mosquito population on the food chain and on the ecological balance. However, elimination of *An. arabiensis *is not expected to affect the food chain significantly since the density of the vector in this area is not high, and other species of mosquitoes (which could serve as alternate prey) are abundant. Also, non-target species are not affected because the SIT is species-specific, and this preserves as much as possible the natural ecosystem. It was clearly demonstrated that the abundance of a non-target anopheline species in the Lake Apastapeque releases in El Salvador, *Anopheles pseudopunctipennis *was not affected by the application of the SIT against the target species *Anopheles albimanus *[11].

The release of large numbers of irradiated sterile mosquitoes into the environment may also raise questions among the local population and effective public information programmes will be required. However, it has to be stressed that the released insects are males, which do not transmit any disease, they are not radioactive, and because they are sterile they do not increase the size of the natural population. Therefore, their release is considered to be essentially benign in terms of environmental impact. Removable of females from the release material is a priority for reasons of efficacy and public health and will be accomplished by the use of a genetic sexing strain in the mass rearing facility.

Several biosafety aspects of handling mosquitoes and the irradiation process must be considered. To prevent the escape of reared mosquitoes, appropriate quarantine containment and precautions will be put in place, as described in the Arthropod Containment Guidelines, Arthropod Containment Level 2 [17]. The irradiator is a fully shielded Nordion 220 cobalt-60 Gammacell located in a secure facility at Soba: it is owned by SAEC and operated by well-trained staff who are required to wear personal dosimeters. The specified gamma radiation dose (70 Gy) currently requires one minute exposure. Dosimetry films are also included with the samples to confirm that the proper radiation dose was received by the mosquitoes.

Malaria cases, and the abundance and genotype of released males, will be monitored indirectly by trapping females and assessing their fertility status. There are currently no trapping methods available to directly monitor sterile male abundance. The abundance of target and non-target mosquitoes, and changes in the number of malaria cases in the release area, will be measured. Any unexpected hazards or outcomes will be reported and responses implemented.

Although capacity building is required mainly to cope with the scientific demands of the project, it is also one of the essential administrative considerations. The project is participating significantly in capacity building in the host institutions and at the field site to ensure sustainability, the appropriate scientific conduct of the study, and to function as equal partners in a collaborative project. The extent to which the project contributes to enhancing local health care and increasing the ability to respond to public health needs through training is one of the most important factors being considered during the development phase of the project.

## Legal aspects

AW-IPM programmes require many activities, which cannot be initiated or carried out without permission from the national authorities, especially from those who are not directly participating in the project. The safety of the project has been assessed by the National Biosafety Framework, which considers primarily environmental risk. The Dam Implementation Unit is responsible for the new Merowe Dam, which is located near the potential release area. The effects of the River height as a result of its operation can be better determined using information they have provided. Obtaining their approval and information will aid in avoiding sensitive issues that could affect the progress of the project. The relatively simple installation of Automated Weather Stations at the field site required permission from the Sudan Meteorology Department, and the acquisition of land for construction required permission from the Northern State Ministry of Planning.

## Social aspects

The success of any public health programme is largely dependent upon education and adequately explaining its aims and objectives to the specific local population [18].

This is especially true for an entomological programme where many people do not understand the disease-vector relationship. For malaria eradication it was observed that the lack of acceptance of residual spraying of DDT in houses was due to the ignorance of villagers as to the goal of the programme [18,19]. Similar difficulties were encountered when radiation-sterilized mosquitoes were released in a village in an attempt to control *Culex fatigans *[20]. Therefore, community inexperience with scientific research is a factor which may increase vulnerability and affect the acceptability of the project.

To encourage community participation in the control of tropical diseases, health education plays a key role, as do the different ways of working with key members of, and organizations in, a community [21]. Community members need to feel that the programme is relevant to their needs and priorities and of potential value to them.

It has been noted that behavioural change, even in well-supported programmes with a high level of participation, was difficult to achieve [22], and the participation of leaders is essential.

Preliminary surveys carried out in Northern State showed that some behavioural practices actually contribute to increasing mosquito density. In spite of the fact that flood pools are the main mosquito breeding sites, man-made breeding sites represent the majority of sites throughout the year. These are mainly pools created for brick manufacturing near the river banks, and are constructed to retain water indefinitely. Irrigation practices are also one of the main sources of breeding sites, although most breeding occurs in riverside pools created when the Nile recedes. Irrigation pools are used in some areas for watering animals, creating permanent breeding sites for the mosquitoes. Villagers use wells as a source of water for purposes other than drinking, creating an additional place for mosquitoes to breed. Other man-made breeding sites include puddles from seepage of broken water pipes and tanks. It is clear that human behaviour in Northern State facilitates year-round breeding of *An. arabiensis*, and so maintains malaria transmission. Efforts in health education should be directed to resolve this behavioural problem so as to keep mosquito density to a minimum level.

### Key issues needed for the development of the SIT feasibility study

• Attraction and involvement of political leaders, relevant stakeholders and related projects, and creation of a good relationship between them and other partners and sponsors

• Addressing the role of mosquito SIT in malaria control and its benefit to the community

• Consideration of the legal issues and ethical questions that arise during the development of the project

• Capacity building at research institutions and at the site of the feasibility study

• Organization of workshops, seminars and public lectures about the project, its objectives and expected outcomes

• Participation of community leaders and the organization of educational programmes that inform the community about the project, and to make the behavioural changes needed to reduce vector populations

## Current activities to overcome some ethical and social problems

Fortunately, the mosquito SIT feasibility study in Northern State has involved all relevant stakeholders through the efforts of the national counterparts and the IAEA experts. They are coordinating efficiently with the IAEA through the strong link of the SAEC. The existing strong collaboration between these institutions and their partners, through the support and enthusiasm of the IAEA and the government, is the first step towards the success of the project.

Political support has been achieved through the formulation of steering committees (ministerial, strategic and operational), holding of regular meetings and seminars, and organization of workshops. These activities are responsible for making political leaders aware of the progress of the project and the essential requirements. Leaders at higher political levels are also involved in one of these committees, ensuring the commitment of the government to this project and its willingness to help and support all its activities. The project is getting considerable political and financial support through these committees, which are working together and meeting regularly.

Scientists working in the feasibility study are addressing the role of the project in malaria control and its benefit to the community through a process of discussion and negotiation; this has already started and will be carried out throughout the course of the project. The discussions always include representatives from relevant stakeholders, such as from executive institutions, the ministry of health, local health authorities, the community and other relevant scientific groups. Furthermore, the discussions also engage international organizations, experts and donor governments to consider financial assistance and capacity building of the host institutions and at the field site.

The project in Sudan is devoted to capacity building. Long- and short-term training abroad for Sudanese researchers on advanced technology have started, and many others are planned. The training is focused on population genetics, GIS/GPS, mass-rearing, and mosquito biology and behaviour. Group training is being carried out during experts' missions for researchers and health personnel of the malaria control programme. Equipment needed to implement use of the collection of GIS data has been installed and is functioning well. For example, four automated weather stations have been installed, and two field stations have been established at the site by the Ministry of Science and Technology. In Khartoum, two insectaries have been established where populations of *An. arabiensis *from the field site have been colonized. Laboratory experiments on these colonies have been started. The insectaries also act as a source of mosquitoes for the colonies at the FAO/IAEA Agriculture and Biotechnology Laboratories in Seibersdorf, Austria.

## Suggestions to overcome other social and ethical problems

Special attention should be paid to the social and ethical questions dealing with the release of irradiated mosquitoes into the environment. While the purpose, technology and effects of SIT are clear and benign to the project participants, one cannot assume that such knowledge is common among those living in the target areas. Therefore, strategies to overcome these disparities must involve scientific exchange and knowledge transfer between scientists involved in the project and this sector of the community. Public lectures, seminars, workshops, video films of successful previous trials and other educational programmes must be organized at different levels to emphasize the biological nature of the SIT.

Although community participation in the project might not be of major importance at the start of the project, community awareness needs to be addressed at an early stage. This could be achieved through community leaders who are essential stimulators; they must understand the objectives of the project and its activities. The orientation of a community leader's involvement should be one of partnership towards mutual education and consensus-building regarding all aspects of the programme. Major steps should be taken to consult the concerned community during both the designing of the project and the course of research and development. Their participation in planning and implementation of the project can provide information regarding their health beliefs and understanding. It can also help in identifying effective methods and the appropriate time for disseminating information about the study and its outcomes

The approach of involving community members requires collective changes in the community's attitudes and behaviour. Changes in knowledge and attitude about the malaria vector and its control can be addressed through education, and unlike behavioural changes can be achieved relatively quickly. For example, behavioural changes that impact mosquito breeding, such as the removal of pools kept for watering animals, may occur neither easily nor rapidly.

## Conclusion

In conclusion, legal, ethical and social problems associated with a mosquito SIT feasibility study can be overcome through rather simple approaches and programmes. Nonetheless, ignoring them at the beginning of a project could lead to adverse effects during subsequent activities, e.g. the release of sterile males. There is a need for good relationships among stakeholders, partners and the community and that they work together to achieve the objectives of the project.

## Competing interests

The authors declare that they have no competing interests.

## Authors' contributions

BBES wrote the paper. All authors contributed to the writing, and read, commented on and approved the chapter.
